# Changes in physical activity and rest-activity circadian rhythm among Hong Kong community aged population before and during COVID-19

**DOI:** 10.1186/s12889-021-10890-x

**Published:** 2021-05-01

**Authors:** Priscilla Ming Yi LEE, Bixia Huang, Gengze Liao, Chi Kuen Chan, Lai-bun Tai, Chun Yuk Jason Tsang, Chi Chiu Leung, Mei-Po Kwan, Lap Ah. Tse

**Affiliations:** 1 JC School of Public Health and Primary Care, The Chinese University of Hong Kong, Hong Kong, China; 2 Pneumoconiosis Clinic, Department of Health, Hong Kong, China; 3 Pneumoconiosis Mutual Aid Association, Hong Kong, China; 4 Stanley Ho Centre for Emerging Infectious Diseases, the Chinese University of Hong Kong, Hong Kong, China; 5 Department of Geography and Resource Management and Institute of Space and Earth Information Science, The Chinese University of Hong Kong, Hong Kong, China

**Keywords:** COVID-19, Physical activity, Circadian rhythm

## Abstract

**Background:**

This study aims to determine the changes in physical activity and actigraphy-measured rest-activity circadian rhythm among Hong Kong community aged population before and during the outbreak of COVID-19.

**Methods:**

This is a three repeated measure population-based cross-sectional study. We recruited community older men aged > 60 years in three periods of the COVID-19 outbreak in Hong Kong, i.e., before the COVID-19 outbreak (2 July 2019–8 January 2020), between the 2nd and 3rd waves of COVID-19 (23 June 2020–9 July 2020), and during the 3rd wave of COVID-19 (15 September 2020–29 September 2020). Participants reported detailed information on their physical activity habits using the International Physical Activity Questionnaire and wore actigraphs continuously for 7 days (168 h). The actigraph data were then transferred to four rest-activity circadian rhythm parameters: midline statistic of rhythm (MESOR), amplitude, acrophase and percent rhythm. Multivariate logistic regression was performed to estimate the association of period effect of COVID-19 on physical activity and rest-activity circadian rhythm parameters.

**Results:**

Among the 242 community older men, 106 (43.8%) of them were recruited before the COVID-19 outbreak, 66 (27.3%) were recruited between the 2nd and 3rd waves of COVID-19, and 70 (28.9%) were recruited during the late phase of the 3rd wave of COVID-19. Compared with those recruited before COVID-19, participants recruited between the 2nd and 3rd waves of COVID-19 had lower physical activity (adjusted odds ratio (AOR) = 2.03, 95% confidence interval (95%CI) =1.05–3.93), MESOR (AOR = 2.05, 95%CI = 1.01–4.18), and amplitude (AOR = 1.91, 95%CI = 0.95–3.83). There was no difference in physical activity or circadian rhythm parameters between subjects recruited before and during the late phase of the 3rd wave.

**Conclusions:**

This study found that the effect of COVID-19 on physical activity and rest-activity circadian rhythm for the community people may be short-term, indicating strong resilience of the community population. Although maintaining physical activity are encouraged for the older adults to sustain good health, a rebound in their physical activity may be a sign for the next wave of outbreak if insufficient social distancing and population protection are facilitated.

**Supplementary Information:**

The online version contains supplementary material available at 10.1186/s12889-021-10890-x.

## Introduction

Coronavirus disease 2019 (COVID-19) is a human-to-human infectious disease which is highly contagious via transmission mainly through respiratory droplets [1]. Up to 27 November 2020, the ongoing pandemic of COVID-19 has led to more than 60,074,174 confirmed cases, with a death toll of greater than 1,416,292 globally [1]. Hong Kong recorded the first confirmed COVID-19 case on 22 January 2020, and Hong Kong Government immediately announced emergency regulations to hamper the spread of COVID-19, including border control and work from home [2]. The outbreak of COVID-19 also awakened the memories of the 2003 Severe Acute Respiratory Syndrome (SARS) epidemic in Hong Kong which promoted many people to conscientiously wear masks and practice social distancing immediately after knowing the spread of a new pathogen in the community. All these intended actions and personal restriction were used to break the chain of infection from the SARS-Cov-2 virus, but on the other hand these control of measures potentially reduced people’s physical activity, dampened their rest-activity circadian rhythms and affected their health.

Circadian rhythm is an endogenous rhythm to help people sustain biological circadian clock periodically in an approximate 24-h cycle [3]. Light exposure and physical activity including showing up at work or performing exercise are the major exogenous time signals that synchronize with the circadian rhythm [3]. The social restrictions during COVID-19 pandemic limited the levels of physical activities and sun light exposure that may adversely influence people’s circadian rhythm and physical health. The World Health Organization (WHO) recommended that older adults aged 65 years old or above should do at least 150–300 min of moderate-intensity aerobic physical activity or do at least 75 min of vigorous-intensity aerobic physical activity per week [4]. However, social restrictions due to COVID-19 resulted in the reduction of physical activity levels among older adults (e.g., less likely to take a walk or do exercises). This physically less active status and the accompanied less robust rest-activity circadian rhythm may pose the aged people at increased risk of diabetes [5], cardiovascular diseases [6, 7] and all-cause mortality [8]. On the other hand, older adults, especially those with pre-existing chronic comorbid conditions (e.g., obesity, heart diseases and diabetes), are more sensitive to changes in the surrounding environment (e.g. infectious agent) [9] and susceptibility to cardiovascular dysfunction and thrombosis [10, 11], which may lead them more likely to develop serious complications from COVID-19 and increase the death rate [10–12].

After the emergence of the global pandemic of COVID-19 in this March, about seven studies reported reduced perceived physical activity levels [13–21], but few of them reported levels of physical activities using objective measurement [16] and none investigated changes in physical activity during different phases of COVID-19. It remains unclear whether the COVID-19 outbreak would affect people’s rest-activity circadian rhythm. In addition, previous meta-analysis study showed that more male patients with COVID-19 than females required intensive treatment per unit admission and had a higher risk of death [22]. The study was commenced in October 2019 aiming at examining whether which male workers with silicosis were more likely to develop sleep deprivation with poor cognition than that of the community subjects. Due to the outbreak of COVID-19 in Hong Kong since January 2020, we had to enroll community subjects intermittently but this provided us an opportunity to recruit participants from different phases of COVID-19 community outbreak. This study aims to determine changes of physical activity levels and actigraphy-measured rest-activity circadian rhythm among Hong Kong community aged men before and during different periods of the COVID-19 outbreak in the city.

## Methods

### Study population and design

**Fig. 1:**
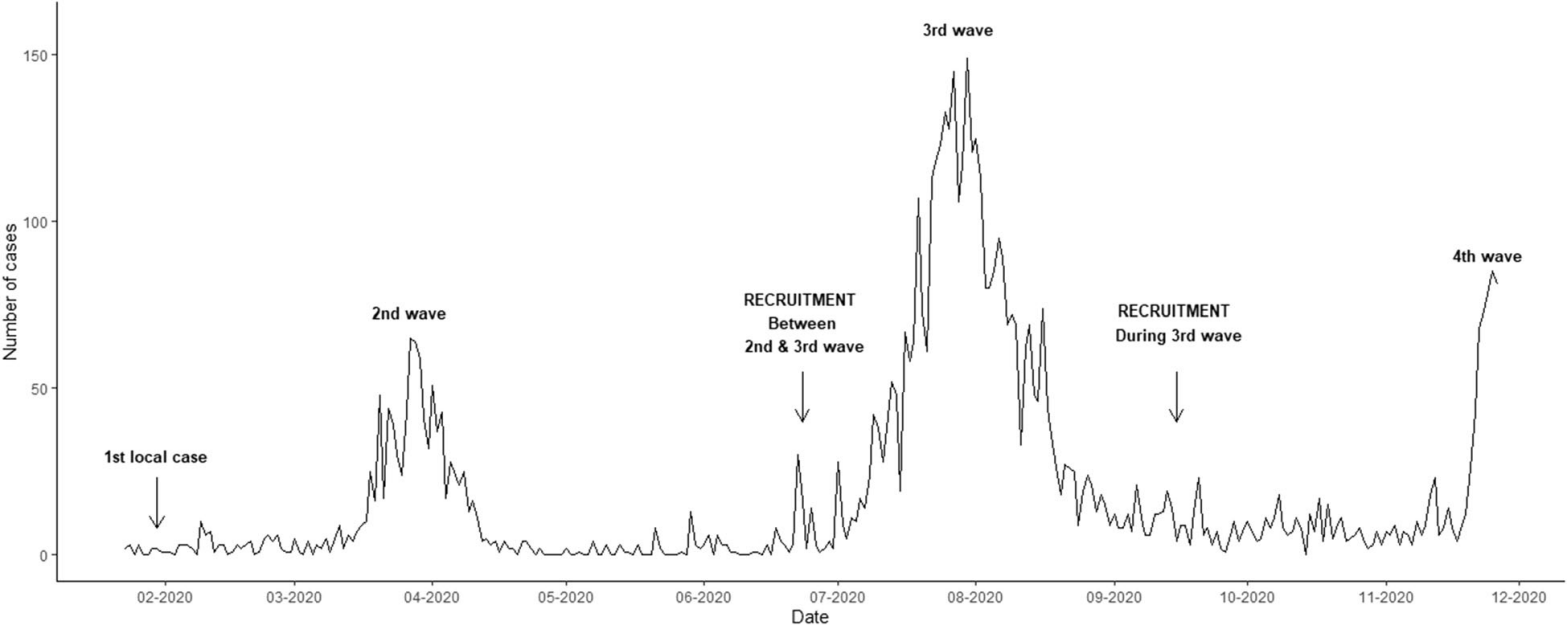
Epidemic curve of confirmed COVID-19 cases in Hong Kong during January 2020 to November 2020 and recruitment time period of 3 batches of study subjects

This is a three repeated measure population-based cross-sectional study. From July 2019 through September 2020, we recruited 3 batches of community controls to compare their patterns of sleep disturbance and circadian rhythm with silicotic patients, and all participants were male. The community controls recruitment was conducted in collaboration with five non-governmental organizations and seven district council members, which located in different area of Hong Kong, including Kwun Tong, Kowloon City, Tsuen Wan, Sham Shui Po and Kwai Tsing Districts. As the recruitment of community subjects covered different periods of the COVID-19 outbreak, this study therefore provides a unique opportunity to investigate the dynamic changes in people’s physical activity and rest-activity circadian rhythm adversely impacted by the outbreak. As shown in Fig. 1, the first recruitment of 106 community subjects was carried out between 2 July 2019 and 1 August 2019, which was before the first positive COVID-19 case was reported in Hong Kong on 23 January 2020. The second recruitment of 66 community subjects were conducted between 23 June 2020 and 9 July 2020, just after the 2nd waves of COVID-19 which had lasted until May 2020 [2]. The third recruitment of 70 community subjects was performed between 15 September 2020 and 29 September 2020, which was during the late phase of the 3rd wave of COVID-19 according to the Hong Kong Government data. Overall, a total of 242 community-dwelling older male participants were included from 3 batches of recruitment covering the period before and during different periods of COVID-19. We excluded participants who have physician diagnosed mental health disorders or medical conditions that prevented them from completing the survey such as hearing problems.

Trained interviewers conducted a face-to-face interview with each participant using standardized questionnaires to obtain information on socio-demographic characteristics, tobacco smoking, alcohol drinking, history of medication uses, physical activity habit and occupational history. We also obtained participants’ anthropometric data through direct measurement including height, weight, and waist circumstance during the interview, where weight measurement was obtained by body composition monitor (TANITA corporation, BC-545 N) according to a standard protocol. Participants were requested to wear light clothes and without shoes during the measurement, and their rest-activity circadian rhythms were measured using actigraphy for seven consecutive 7 days (168 h). This study was approved by the Joint Chinese University of Hong Kong-New Territories East Cluster Clinical Research Ethics Committee, and all participants signed written informed consent forms before the survey was conducted.

### Outcome measurements and procedure

#### Physical activity

We adopted the validated International Physical Activity Questionnaire (IPAQ)-short form to collect detailed information of participants’ physical activity habit, including the frequency and duration of vigorous-intensity and moderate-intensity physical activity, and walking during the past week. We further recorded the types of their physical activity such as hiking, weight lifting, jogging, and tai chi. We matched the physical activity information of our participants with metabolic equivalents (METS) levels obtained from the 2000 and 2011 compendium of physical activities [23, 24], and calculated their total weekly physical activity levels as duration (minutes) × frequency per week (days) × METS intensity. We further categorized their physical activity levels into low and high according to the median METS of our population. A METS refers to the resting metabolic rate, which is the amount of oxygen consumed while sitting at rest [25].

#### Rest-activity circadian rhythm

Each participant was requested to wear a GENEActiv Original (Activinsights Company, UK) device on his non-dominant wrist continuously for 7 days (168 h) with measurement frequency of 100 Hz and a sampling rate of 1 min. The assessment of circadian rhythm parameters had been described previously [26]. Briefly, the actigraphy detected and recorded individual movements in three mutually vertical axes (x, y, and z). A gravity-subtracted sum of vector magnitudes (SVM) was automatically calculated with data of these three axes and a formula defined by the manufacturer: SVM = [(x^2^ + y^2^ + z^2^)½ - 1 g] [27]. The SVM data were then imported into the Chronos-Fit program (v. 1.06) [28] to facilitate computing four rest-activity circadian rhythm parameters, namely midline statistic of rhythm (MESOR), amplitude, acrophase and percent rhythm by an extended cosine model [29]. MESOR refers to the adjusted mean levels of the rhythm. Amplitude refers to the magnitude of the rhythm cycle (i.e. the distance between the peak and MESOR). Acrophase refers to the time in the cycle of the daily peak rhythm; earlier time of peak rhythm suggests an advanced acrophase and later peak rhythm time suggests a delay acrophase. Percent rhythm refers to the percentage of variation in the data that is explained by the fitted model; a low percent rhythm suggests a dampened rhythm [30]. We also coded the SVM into one of three intensity categories: sedentary (SVM < 158.5), light (SVM = 158.5–261.8), moderate or vigorous activity (SVM > 261.8) according to previously validated cut-off for GENEActiv accelerometers among adults (mean aged =59.6 ± 5.5 years) [31].

Non-wearing time was determined by reviewing the SVM readings outputted from the GENEActiv software and the self-reported sleep log data collected from the interviewees. The non-wearing periods should present low and steady SVM readings, and we excluded these data from the calculation of the parameters. Only participants with a total length of wearing time more than 120 h (5/7 consecutive days of 168 h) that covered weekends records were included in the study. All rest-activity circadian rhythm parameters were stratified based on their median distribution, and categorized into low and high levels for MESOR (< 270.25/> 270.25), amplitude (< 132.56/> 132.56) and percent rhythm (< 18.09/> 18.09), and for acrophase it was classified as advanced and delayed acrophase (peak activity before 2:04 pm / after 2:04 pm).

### Statistical analysis

We performed independent t-tests and chi-square test to compare the differences in basic sociodemographic characteristics, levels of physical activity and circadian rhymes for community subjects completed the interview during different periods of COVID-19 (i.e., before COVID-19, between the 2nd and 3rd waves of COVID-19, and during the late phase of the 3rd waves of COVID-19), using the category “before COVID-19” as the reference group. We performed multivariate unconditional logistic regression model to estimate the odds ratios (OR) and the 95% confidence intervals (95%CI) to examine the period effect of COVID-19 (i.e., before COVID-19, between the 2nd and 3rd waves of COVID-19, and during the late phase of the 3rd waves of COVID-19) on participants’ physical activity levels (METS/minutes) (low/high) and rest-activity circadian rhythm parameters [i.e. MESOR (low/high), amplitude (low/high), acrophase (advanced/delayed) and percent rhythm (low/high)] before and during different periods of the outbreak, using the high-level category of physical activity, MESOR, amplitude and percent rhythm, and advanced acrophase as the reference group. Potential confounders included in the multivariate logistic regression were age at recruitment (continuous), educational attainment (primary education or below, secondary education or above), employment status (retired, full-time/part-time job), and body mass index (BMI) [body weight (kg)/height^2^(m^2^)] [underweight/normal weight (BMI < 25), overweight, obesity (BMI > 25]. All statistical analyses were conducted with SPSS 26.0 for Windows (SPSS, Chicago, IL, USA), and a two-sided *p*-value of less than 0.05 was considered statistically significant.

## Results

### Selected characteristics of study population

A total of 242 older men were recruited from the community with 3 batches during the period from 2 July 2019 to 29 September 2020 (i.e., before the COVID-19 outbreak, between the 2nd and 3rd waves of COVID-19 and during the late phase of the 3rd wave of COVID-19), and their basic characteristics are summarized in Table 1. Compared with the older men recruited before the COVID-19 outbreak and during the 3rd wave of COVID-19, more older men recruited between the 2nd and 3rd waves of COVID-19 were unemployed or retired. There was no statistically significant difference in age, educational attainment, marital status, tobacco and alcohol consumption habit and BMI when comparing the community subjects recruited before the COVID-19 outbreak, between the 2nd and 3rd waves of COVID-19 and during the late phase of the 3rd wave of COVID-19.

**Table 1:** Distribution of selected characteristics by time of COVID-19 outbreak among 242 Hong Kong older men

	Before COVID-19 outbreak *N* = 106 *N*(%)	Between 2nd and 3rd waves of COVID-19 *N* = 66 *N*(%)	During 3rd wave of COVID-19 *N* = 70 *N*(%)	*p*-value
**Age**, mean + SD	73.67 + 8.76	73.37 + 6.36	71.43 + 8.28	0.173
**Education attainment**				0.894
Primary education or below	60 (56.6)	36 (54.5)	41 (58.6)	
Secondary education or above	46 (43.4)	30 (45.5)	29 (41.4)	
**Marital status**				0.774
Married	84 (81.6)	50 (78.1)	58 (82.9)	
Single/widow/divorce	19 (18.4)	14 (21.9)	12 (17.1)	
**Employment status**				0.096
Retired/unemployed	87 (82.9)	60 (90.9)	54 (77.1)	
Full-time/Part-time	18 (17.1)	6 (9.1)	16 (22.9)	
**Smoking**				0.409
Never smoke	56 (52.8)	28 (42.4)	35 (50.0)	
Smoker/former smoker	50 (47.2)	38 (57.6)	35 (50.0)	
**Alcohol consumption**				0.181
Non-drinker	72 (67.9)	43 (65.2)	55 (78.6)	
Drinker/former drinker	34 (32.1)	23 (34.8)	15 (21.4)	
**BMI**				0.888
Underweight/Normal weight	67 (63.2)	38 (57.6)	45 (65.2)	
Overweight	32 (30.2)	22 (33.3)	20 (29.0)	
Obesity	7 (6.6)	6 (9.1)	4 (5.8)	

*Abbreviations*: *1st* first, *2nd* second, *3rd* third, *BMI* body mass index

**Fig. 2:**
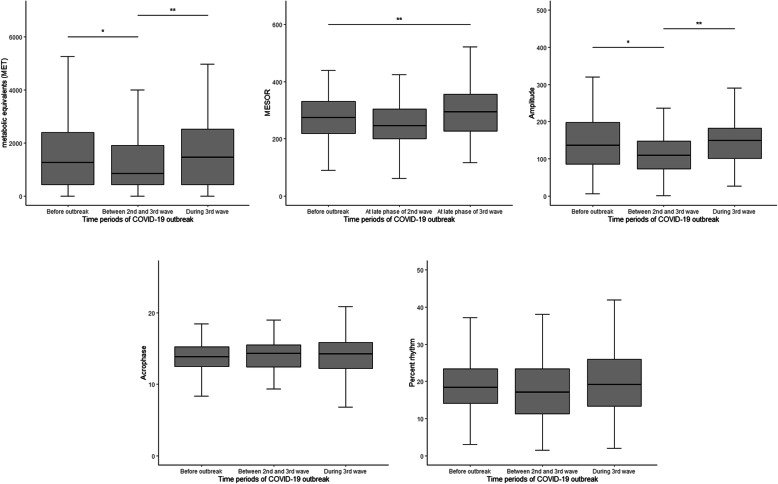
Distribution of metabolic equivariate (MET) according to the time of COVID-19 outbreak

Figure 2 compares the levels of physical activity (MET/minutes) among participants recruited during different periods of COVID-19, and showed that the level of physical activity for participants recruited between the 2nd and 3rd waves of COVID-19 were significantly lower than those recruited before the COVID-19 outbreak or during the 3rd waves of COVID-19. The decreased levels of physical activity for participants recruited between and during the 2nd and 3rd waves of COVID-19 were mainly from outdoor activities such as running, swimming and hiking (Supplementary Table 1). In addition to the actigraphy measurement, 217 participants agreed to wear an accelerometer continuously for five to 7 days and have sufficient actigraph data. As shown in Fig. 2, compared with those recruited before COVID-19, participants recruited between the 2nd and 3rd waves of COVID-19 had lower levels of MESOR and amplitude, but these measurements rebounded for participants recruited during the late phase of the 3rd wave of COVID-19 to the levels similar to those measured before COVID-19.

### Selected characteristics with physical activity levels and rest-activity circadian rhythm

Table 2 presents the odds ratio (OR) and 95% confidence interval (95%CI) resulting from univariate logistic regression for the association of basic characteristics with physical activity levels (METS/minutes) and rest-activity circadian rhythm parameters. Being older was associated with low levels of MESOR (OR = 1.04, 95%CI = 1.00–1.08) and amplitude (OR = 1.07, 95%CI = 1.03–1.11), as well as advanced acrophase (OR = 1.04, 95%CI = 1.00–1.07). Participants with lower educational attainment tended to have advanced acrophase (OR = 2.85, 95%CI = 1.64–4.96), while participants who were already retired or unemployed were associated with lower MESOR (OR = 3.89, 95%CI = 1.74–8.71) and amplitude (OR = 2.83, 95%CI = 1.32–6.06). Moreover, participants who were overweight had lower levels of physical activity (95%CI = 1.07–3.34) and MESOR (95%CI = 1.00–3.32) than those with normal BMI (Table 2).

**Table 2:** Association of selected characteristics with physical activity levels and rest-activity circadian rhythm parameters

	Physical activity			Rest-activity circadian rhythm											
	MET/minutes			MESOR			Amplitude			Acrophase			Percent rhythm		
	Low *N* = 132 *N*(%)	High *N* = 110 *N*(%)	Crude OR (CI)	Low *N* = 109 *N*(%)	High *N* = 108 *N*(%)	Crude OR (CI)	Low *N* = 109 *N*(%)	High *N* = 108 *N*(%)	Crude OR (CI)	Advanced *N* = 109 *N*(%)	Delayed *N* = 108 *N*(%)	Crude OR (CI)	Low *N* = 109 *N*(%)	High *N* = 108 *N*(%)	Crude OR (CI)
**Age**	73.13 + 8.21	72.71 + 7.91	1.01 (0.98–1.04)	73.96 + 7.72	71.64 + 7.74	**1.04 (1.00–1.08)**	74.71 + 7.54	70.89 + 7.62	**1.07 (1.03–1.11)**	73.83 + 7.54	71.77 + 7.62	**1.04 (1.00–1.07)**	72.57 + 7.74	73.03 + 7.89	0.99 (0.96–1.03)
**Education attainment**															
Primary education or below	74 (56.1)	63 (57.3)	0.95 (0.57–1.59)	67 (62.0)	53 (48.6)	**0.58 (0.34–0.99)**	60 (55.0)	60 (55.6)	0.98 (0.57–1.67)	74 (67.9)	46 (42.6)	**2.85 (1.64–4.96)**	60 (55.0)	60 (55.6)	0.98 (0.57–1.67)
Secondary education or above	58 (43.9)	47 (42.7)	1.00	41 (38.0)	56 (51.4)	1.00	49 (45.0)	48 (44.4)	1.00	35 (32.1)	62 (57.4)	1.00	49 (45.0)	48 (44.4)	1.00
**Marital status**															
Married	109 (82.6)	83 (75.5)	1.00	82 (75.2)	90 (83.3)	1.00	85 (81.0)	87 (81.3)	1.00	87 (81.3)	85 (81.0)	1.00	81 (76.4)	91 (84.3)	1.00
Single/ widow/ divorce	20 (15.2)	25 (22.7)	0.61 (0.32–1.17)	22 (20.2)	18 (16.7)	1.34 (0.67–2.68)	20 (19.3)	20 (18.7)	1.02 (0.51–2.04)	20 (18.7)	20 (19.0)	0.98 (0.49–1.94)	25 (23.6)	15 (13.9)	1.87 (0.92–3.80)
**Employment status**															
Retired/unemployed	107 (81.7)	94 (85.5)	0.76 (0.38–1.51)	100 (91.7)	80 (74.1)	**3.89 (1.74–8.71)**	98 (89.9)	82 (75.9)	**2.83 (1.32–6.06)**	89 (81.7)	91 (84.3)	0.83 (0.41–1.69)	95 (87.2)	85 (78.7)	1.84 (0.89–3.80)
Full-time/Part-time	24 (18.3)	16 (14.5)	1.00	9 (8.3)	28 (25.9)	1.00	11 (10.1)	26 (24.1)	1.00	20 (18.3)	17 (15.7)	1.00	14 (12.8)	23 (21.3)	1.00
**Smoking**															
Never smoke	62 (47.0)	57 (51.8)	1.00	50 (45.9)	56 (51.9)	1.00	53 (48.6)	53 (49.1)	1.00	51 (46.8)	55 (50.9)	1.00	49 (45.0)	57 (52.8)	1.00
Smoker/former smoker	70 (53.0)	53 (48.2)	1.21 (0.73–2.02)	59 (54.1)	52 (48.1)	1.27 (0.75–2.17)	56 (51.4)	55 (50.9)	1.02 (0.60–1.73)	58 (53.2)	53 (49.1)	1.18 (0.69–2.01)	60 (55.0)	51 (47.2)	1.37 (0.80–2.33)
**Alcohol consumption**															
Non-drinker	93 (70.5)	77 (70.0)	1.00	74 (67.9)	76 (70.4)	1.00	76 (69.7)	74 (68.5)	1.00	71 (65.1)	79 (73.1)	1.00	76 (69.7)	74 (68.5)	1.00
Drinker/former drinker	39 (29.5)	33 (30.0)	0.78 (0.56–1.70)	35 (32.1)	32 (29.6)	1.12 (0.63–2.00)	33 (30.3)	34 (31.5)	0.95 (0.53–1.68)	38 (34.9)	29 (26.9)	1.46 (0.82–2.60)	33 (30.3)	34 (31.5)	0.95 (0.53–1.68)
**BMI**															
Underweight/ Normal weight	72 (55.0)	78 (70.9)	1.00	61 (56.5)	74 (68.5)	1.00	64 (58.7)	71 (65.7)	1.00	70 (64.8)	65 (60.2)	1.00	62 (57.4)	73 (67.6)	1.00
Overweight	47 (35.9)	27 (24.5)	**1.87 (1.07–3.34)**	39 (36.1)	26 (24.1)	**1.82 (1.00–3.32)**	34 (31.2)	31 (28.7)	1.22 (0.67–2.20)	31 (28.7)	34 (31.5)	0.85 (0.47–1.53)	35 (32.4)	30 (27.8)	1.37 (0.76–2.49)
Obesity	12 (9.2)	5 (4.5)	2.60 (0.87–7.74)	8 (7.4)	8 (7.4)	1.21 (0.43–3.42)	10 (9.2)	6 (5.6)	1.85 (0.64–5.37)	7 (6.5)	9 (8.3)	0.72 (0.25–2.05)	11 (10.2)	5 (4.6)	2.59 (0.85–7.86)

*Abbreviations*: *OR* odds ratio, *CI* confidence intervals, *BMI* body mass index

Table 3 shows the period effect of COVID-19 on the levels of physical activity and rest-activity circadian rhythm parameters. Compared to participants recruited before the COVID-19 outbreak, participants recruited between the 2nd and 3rd waves of COVID-19 were more likely to have lower levels of physical activity (OR = 1.86, 95%CI = 0.98–3.51), MESOR (OR = 2.18, 95%CI = 1.11–4.29) and amplitude (OR = 2.03, 95%CI = 1.04–3.96), and the association remained unchanged after the adjustment of age, educational attainment, employment status and BMI (physical activity: adjusted OR = 2.03, 95%CI = 1.05–3.93); MESOR: adjusted OR = 2.05, 95%CI = 1.01–4.18); amplitude: adjusted OR = 1.91, 95%CI = 0.95–3.83). A similar finding was obtained for the association between COVID-19 periods and percent rhythm (adjusted OR = 1.46, 95%CI = 0.70–2.67), although it was not significant (Table 3). In addition, we stratified SVM into sedentary, light, and moderate or vigorous activity according to previously validated cut-off scale [31]. Compared to participants recruited before the COVID-19 outbreak, more participants recruited between the 2nd and 3rd waves of COVID-19 tended to have sedentary (adjusted OR = 1.56, 95%CI = 0.48–5.09) and light activities (adjusted OR = 1.66, 95%CI = 0.80–3.42) (Supplementary Table 2), although they were not statistically significant. Physical activity levels and rest-activity circadian rhythm parameters obtained during the late phase of the 3rd were rebounded to the levels measured before the outbreak of COVID-19.

**Table 3:** Association of period of COVID-19 outbreak with physical activity levels and rest-activity circadian rhythm parameters

	Physical activity				Rest-activity circadian rhythm ^b^															
	MET/minutes				MESOR				Amplitude				Acrophase				Percent rhythm			
**Period of COVID-19**	Low *N* = 132 *N*(%)	High *N* = 110 *N*(%)	Crude OR (CI)	Adjusted OR (CI) ^a^	Low *N* = 109 *N*(%)	High *N* = 108 *N*(%)	Crude OR (CI)	Adjusted OR (CI) ^a^	Low *N* = 109 *N*(%)	High *N* = 108 *N*(%)	Crude OR (CI)	Adjusted OR (CI) ^a^	Advanced *N* = 109 *N*(%)	Delayed *N* = 108 *N*(%)	Crude OR (CI)	Adjusted OR (CI) ^a^	Low *N* = 109 *N*(%)	Hig *N* = 108 *N*(%)	Crude OR (CI)	Adjusted OR (CI) ^a^
Before outbreak	55 (41.7)	51 (46.4)	1.00	1.00	44 (40.4)	48 (44.4)	1.00	1.00	44 (40.4)	48 (44.4)	1.00	1.00	49 (45.0)	43 (39.8)	1.00	1.00	45 (41.3)	47 (43.5)	1.00	1.00
Between 2nd and 3rd waves of COVID-19	44 (33.3)	22 (20.0)	1.86 (0.98–3.51)	**2.03 (1.05–3.93)**	40 (36.7)	20 (18.5)	**2.18 (1.11–4.29)**	**2.05 (1.01–4.18)**	39 (35.8)	21 (19.4)	**2.03 (1.04–3.96)**	1.91 (0.95–3.83)	28 (25.7)	32 (29.6)	0.77 (0.40–1.47)	0.75 (0.38–1.49)	35 (32.1)	25 (23.1)	1.46 (0.76–2.82)	1.37 (0.70–2.67)
During 3rd wave of COVID-19	33 (25.0)	37 (33.6)	0.83 (0.45–1.51)	0.83 (0.44–1.56)	25 (22.9)	40 (37.0)	0.68 (0.36–1.30)	0.72 (0.36–1.43)	26 (23.9)	39 (36.1)	0.73 (0.38–1.38)	0.80 (0.41–1.58)	32 (29.4)	33 (30.6)	0.85 (0.45–1.61)	0.82 (0.42–1.61)	29 (26.6)	36 (33.3)	0.84 (0.45–1.59)	0.83 (0.43–1.60)

*Abbreviations*: *OR* odds ratio, *CI* confidence intervals, *1st* first, *2nd* second, *3rd* third, *MESOR* Midline statistic of rhythm

^a^ Adjusted for the age at interview, sex, education attainment, employment status, and BMI. ^b^ All rest-activity rhythms parameters were stratified based on their median distribution

## Discussion

This study observed lower levels of physical activity and rest-activity circadian rhythm parameter MESOR and amplitude among participants recruited between the 2nd and 3rd waves of COVID-19 than those among participants recruited before the COVID-19 outbreak, but these measurements rebounded among participants recruited during the late phase of the 3rd wave of COVID-19. Similar to the previous studies, older age, lower educational attainment, unemployment status and being overweight were also related to lower levels of physical activity and rest-activity circadian rhythm parameters [32, 33].

In late March of 2020, Hong Kong experienced the second wave of COVID-19 and the government immediately announced restrictive measures to control the spread of COVID-19, including closing its border to all incoming non-residents from overseas, banning any gathering of more than four people, or requiring restaurants to set their Tables 1.5 m apart from each other [2]. As older adults tended to have more serious symptoms of COVID-19 [12], they were advised to stay at home and limit their outdoor activities, which consequently led them to have almost no or very low levels of physical activity. This is consistent with our finding that participants recruited between the 2nd and 3rd waves of COVID-19 had significantly lower levels of physical activities and rest-activity circadian rhythm parameters “MEOSR” and “amplitude.” The dampened rest-activity circadian rhythm and reduced levels of physical activity might adversely affect older adults’ physical and mental health, including hypertension, diabetes, overweight, depression and anxiety [5–7, 13, 34], which may in turn also affect their quality of life.

In early July of 2020, Hong Kong experienced the 3rd wave of COVID-19 and the daily numbers of local confirmed new cases reached the highest since the outbreak. The Hong Kong Government thus announced further restrictive measures such as mandatory mask-wearing in all public areas (both indoor and outdoor), or temporarily closed the gyms, beauty salons, bars and other public venues [2]. Intriguingly, the levels of physical activity or rest-activity circadian rhythm resumed to the normal levels before the COVID-19 outbreak. Our study found that older people in Hong Kong had great resilience in their levels of physical activity and rest-activity circadian rhythm under the COVID-19 control measures. In addition, we observed that there was a reduction of numbers of running, swimming or going to gym room for exercise during the during the late phase of the 3rd waves of COVID-19, but an increased in numbers of stretching, speed walking or other activities. It indicated that older adults regained their physical activities levels by other activities. Home-based physical activity including stationary aerobic exercise or weight/strength training should be encouraged for those people who have been ordered or advised to stay at home, especially for countries that implemented lockdowns or mandated that infected people being quarantine/self-isolate. The restrictive regulations from the government such as mandatory mask-wearing in all public places may also benefit older adults in maintaining their physical activity levels (for instance, they may take a regular walk or exercises) in order to prevent worsened physical and mental health outcomes.

### Strength and limitation

This is a cross-sectional study that recruited 3 batches of subjects during three different periods of the COVID-19 outbreak in Hong Kong. It dynamically captured the differences in participants’ physical activity levels and robustness of rest-activity circadian rhythm measured by validated IPAQ and objective actigraphy that have never been reported in previous studies. This is the major merit of the study. However, as our study subjects were recruited based on voluntary participation and selection bias may be a concern. Also, as our sample size was small and the 95%CI of some categories are broader, the observed results may be due to chance. We were also aware that physical activities levels could be stratified into three categories (i.e. low, moderate, high) according to the IPAQ instruction [35], however, small sample size limited a further in-depth analysis. In addition to the selection bias, we only recruited community older men as to compare with the silicotic patients recruited in our original study. Women are generally less physically active than men [36], and thus this study may not be generalize to female older adults. Nevertheless, COVID-19 is new and there are many uncertainties with this global infectious disease. Any dynamic data covering different periods of the pandemic would have considerable potential for advancing our knowledge and understanding of this emerging infectious disease and will be valuable.

## Conclusion

In conclusion, the study found that the effect of COVID-19 on physical activity and rest-activity circadian rhythm for the community people may be short-term, indicating strong resilience of the community population. Although maintaining physical activity is encouraged for older adults to sustain good health, a rebound in physical activity may be a sign for the next wave of outbreak if insufficient social distancing and population protection are facilitated. The findings may have important implications on formulation of social distancing strategies especially since more waves of coronavirus infection are likely to occur until most members of the public get vaccinated.

## Supplementary Information

**Additional file 1: Table S1.** Distribution of type of physical activities by the time of COVID-19 outbreak among 242 Hong Kong older men. **Table S2.** Association between period of COVID-19 outbreak and physical activity levels according to sum of vector magnitudes.

## Data Availability

The results and materials described in the article, and the relevant data related to physical activity and rest-activity circadian rhythm that used in this article, are available from the corresponding author on reasonable request.

## Abbreviations

COVID-19: Coronavirus disease 2019
SARS: Severe Acute Respiratory Syndrome
WHO: World Health Organization
IPAQ: International Physical Activity Questionnaire
METS: Metabolic equivalents
SVM: Sum of vector magnitudes
MESOR: Midline statistic of rhythm
OR: Odds ratio
95%CI: 95% confidence intervals
BMI: Body mass index
