# Supplementary material for: Changes in physical activity and rest-activity circadian rhythm among Hong Kong community aged population before and during COVID-19
Source: BMC Public Health. 2021 May 1;21:836. doi: 10.1186/s12889-021-10890-x (PMC8087874; doi:10.1186/s12889-021-10890-x)
Supplement: Supplementary file 1 — Additional file 1: Table S1. Distribution of type of physical activities by the time of COVID-19 outbreak among 242 Hong Kong older men. Table S2. Association between period of COVID-19 outbreak and physical activity levels according to sum of vector magnitudes. [file 12889_2021_10890_MOESM1_ESM.docx]

Supplementary table 1 Distribution of type of physical activities by the time of COVID-19 outbreak among 242 Hong Kong older men

|  | Before COVID-19 outbreak  N= 106  N(%) | Between 2^nd^ and 3^rd^ waves of COVID-19  N= 66  N(%) | During 3^rd^ wave of COVID-19  N= 70  N(%) |
| --- | --- | --- | --- |
| **Types of physical activities** |  |  |  |
| Running | 10 (9.4) | 5 (7.6) | 5 (7.1) |
| Swimming | 8 (7.4) | 2 (3.0) | 1 (1.4) |
| Hiking | 12 (11.3) | 4 (6.1) | 5 (7.1) |
| Speed walking | 6 (5.7) | 4 (6.1) | 6 (8.6) |
| Workout/gym room | 6 (5.7) | 5 (7.6) | 3 (4.3) |
| Stretching | 11 (10.4) | 8 (12.1) | 11 (15.7) |
| Others | 17 (16.0) | 6 (9.1) | 10 (14.3) |

Supplementary table 2. Association between period of COVID-19 outbreak and physical activity levels according to sum of vector magnitudes

|  |  | | **Physical activity** | | | | | | |
| --- | --- | --- | --- | --- | --- | --- | --- | --- | --- |
|  |  | |  |  | | Sedentary | | Light | |
| **Period of COVID-19** | Sedentary  n=14 n(%) | Light   n=75 n(%) | | | Moderate or vigorous n=108 n(%) | Crude OR(CI) | Adjusted OR (CI) ^a^ | Crude OR(CI) | Adjusted OR (CI) ^a^ |
| Before outbreak | 8 (8.7) | 34 (37.0) | | | 50 (54.3) | 1.00 | 1.00 | 1.00 | 1.00 |
| Between 2^nd^ and 3^rd^ waves of COVID-19 | 6 (10.0) | 30 (50.0) | | | 24 (40.0) | 1.56 (0.49-5.01) | 1.56 (0.48-5.09) | 1.84 (0.92-3.67) | 1.66 (0.80-3.42) |
| During 3^rd^ wave of COVID-19 | 3 (4.5) | 20 (30.8) | | | 42 (64.6) | 0.45 (0.11-1.79) | 0.33 (0.06-1.65) | 0.70 (0.35-1.39) | 0.79 (0.38-1.61) |

Abbreviations: OR, odds ratio; CI, confidence intervals, 1^st^=first, 2^nd^=second, 3^rd^=third.

^a^ Adjusted for the age at interview, sex, education attainment, employment status, and BMI.
